# Long-Term Dynamic Changes in Hybrid Immunity over Six Months after Inactivated and Adenoviral Vector Vaccination in Individuals with Previous SARS-CoV-2 Infection

**DOI:** 10.3390/vaccines12020180

**Published:** 2024-02-10

**Authors:** Nungruthai Suntronwong, Sitthichai Kanokudom, Chompoonut Auphimai, Thanunrat Thongmee, Suvichada Assawakosri, Preeyaporn Vichaiwattana, Ritthideach Yorsaeng, Thaneeya Duangchinda, Warangkana Chantima, Pattarakul Pakchotanon, Pornjarim Nilyanimit, Donchida Srimuan, Thaksaporn Thatsanathorn, Natthinee Sudhinaraset, Nasamon Wanlapakorn, Yong Poovorawan

**Affiliations:** 1 Center of Excellence in Clinical Virology, Faculty of Medicine, Chulalongkorn University, Bangkok 10330, Thailand; nungruthai.s@chula.ac.th (N.S.); sitthichai.k@chula.ac.th (S.K.); chompoonut.bit@gmail.com (C.A.); thanunrat.t@chulahospital.org (T.T.); 6273005030@student.chula.ac.th (S.A.); preeyaporn.vic@chulahospital.org (P.V.); ritthideach.yor@gmail.com (R.Y.); mim_bhni@hotmail.com (P.N.); donchida.s@gmail.com (D.S.); thaksapohnl@hotmail.com (T.T.); dr_natthinee@hotmail.com (N.S.); nasamon.w@chula.ac.th (N.W.); 2 Center of Excellence in Osteoarthritis and Musculoskeleton, Faculty of Medicine, Chulalongkorn University, King Chulalongkorn Memorial Hospital, Thai Red Cross Society, Bangkok 10330, Thailand; 3 Molecular Biology of Dengue and Flaviviruses Research Team, National Center for Genetic Engineering and Biotechnology (BIOTEC), National Science and Development Agency, NSTDA, Pathum Thani 12120, Thailand; thaneeya.dua@biotec.or.th (T.D.); mk_pk@msn.com (P.P.); 4 Division of Dengue Hemorrhagic Fever Research, Faculty of Medicine Siriraj Hospital, Mahidol University, Bangkok 10700, Thailand; warangkana_ch1@hotmail.com; 5 Siriraj Center of Research Excellence in Dengue and Emerging Pathogens, Faculty of Medicine Siriraj Hospital, Mahidol University, Bangkok 10700, Thailand; 6 The Royal Society of Thailand (FRS(T)), Sanam Sueapa, Dusit, Bangkok 10330, Thailand

**Keywords:** hybrid immunity, CoronaVac, ChAdOx1, SARS-CoV-2, omicron, long-term follow-up, durability, previous infection, COVID-19

## Abstract

Numerous studies have largely focused on short-term immunogenicity in recovered individuals post mRNA vaccination. However, understanding the long-term durability, particularly in inactivated and adenoviral vectored vaccines, remains limited. We evaluated antibody responses, omicron variant neutralization, and IFN-γ responses in 119 previously infected individuals vaccinated with CoronaVac or ChAdOx1 up to six months post-vaccination. Both vaccines elicited robust immune responses in recovered individuals, surpassing those who were infection-naïve, and these persisted above pre-vaccination levels for six months. However, antibody levels declined over time (geometric mean ratio (GMR) = 0.52 for both vaccines). Notably, neutralizing activities against omicron declined faster in ChAdOx1 (GMR = 0.6) compared to CoronaVac recipients (GMR = 1.03). While the first dose of ChAdOx1 adequately induced immune responses in recovered individuals, a second dose demonstrated advantages in omicron variant neutralization and slower decline. Although both vaccines induced T cell responses, the median IFN-γ level at six months returned to pre-vaccination levels. However, more individuals exhibited reactive T cell responses. Extending the interval (13–15 months) between infection and vaccination could enhance antibody levels and broaden neutralization. Together, these findings demonstrate a robust humoral and cellular response that was sustained for at least six months after vaccination, thus guiding optimal vaccination strategies based on prior infection and vaccine platforms.

## 1. Introduction

The Coronavirus Disease 2019 (COVID-19) pandemic has affected nearly 800 million confirmed cases globally, resulting in seven million reported deaths as of 20 November 2023 [1]. COVID-19 vaccines were developed to mitigate the impact of the disease and reduce its severity. As of 17 November 2023, more than 13.5 billion COVID-19 vaccine doses have been administered worldwide [1]. In Thailand, a national COVID-19 immunization program commenced on 28 February 2021, which primarily employed the CoronaVac (Sinovac Life Sciences Co., LTD, Beijing, China) and ChAdOx1 nCoV-19 (recombinant; Oxford-AstraZeneca) vaccines during its initial phase. CoronaVac, an inactivated whole virus COVID-19 vaccine adjuvanted with aluminum hydroxide, has seen over three billion doses administered across 62 countries, with a primary focus on low or middle-income regions. Clinical trials reported an efficacy ranging from 50 to 84% in protecting against symptomatic COVID-19 [2]. Additionally, the ChAdOx1 nCoV-19 vaccine, a replication-deficient chimpanzee adenoviral vector containing the SARS-CoV-2 spike antigen, accounts for three billion doses distributed across more than 170 countries worldwide, and it has shown efficacy rates of up to 70.4% in preventing symptomatic COVID-19 [3].

Recent findings indicate that individuals recovering from SARS-CoV-2 infection typically develop robust humoral and T cell-mediated immune responses that are associated with disease protection [4]. Long-term monitoring has revealed a modest decline in SARS-CoV-2 antibody levels, and this was observed between 5 to 8 months post-infection [5]. B cell memory to SARS-CoV-2 can remain detectable for up to eight months following natural infection [5,6]. Although the T cell-mediated immune response appears to persist for several months, where it lasts longer than the detectable antibodies in recovered patients, a decrease in memory T cells with an initial half-life of 3 to 5 months has been reported [5,6]. Emerging evidence has suggested that vaccination among individuals with prior SARS-CoV-2 infection could sustain protective immunity and offer additional protection against symptomatic reinfection and severe outcomes [7,8].

The combination of natural immunity and vaccine-induced protection, termed hybrid immunity, presents a notably stronger and broader immune response, thereby surpassing the effects of either infection or vaccination alone [9]. However, existing evidence has largely focused on the early response following mRNA vaccination among previously infected individuals [10,11,12,13]. Few studies have evaluated the impacts of inactivated and vector-based vaccines [14,15,16]. Additionally, most investigations have focused on short-term immunogenicity, while long-term evaluations remain limited.

The surge of omicron variants, marked by substantial mutations, has the potential to increase virus transmissibility and reduce susceptibility to neutralization by antibodies induced through natural infection or vaccination [17]. Additionally, the documented progressive decline in the humoral immune response is well established in vaccinated individuals and those previously infected with SARS-CoV-2 [18,19]. Therefore, it is crucial to comprehend the trajectory and long-term durability of the immune response and its potential for neutralizing the omicron variant. However, the longitudinal data characterizing the extended dynamics of antibodies, neutralization capability, and T cell responses post-immunization with various vaccines—specifically comparing those currently in use, such as inactivated and adenoviral vectored vaccines, in individuals with prior infection—remain unclear.

In this study, we aimed to assess the dynamic changes and decline in humoral and T cell immune responses over a six-month period following the administration of either inactivated (CoronaVac) or adenoviral vector-based vaccines (ChAdOx1 nCoV-19) among individuals previously infected with SARS-CoV-2. Additionally, we quantified the impact of the time interval between infection and vaccination on neutralization against pre-omicron and omicron variants, as well as of T cell responses. These analyses provide insights into the durability of immune responses induced by inactivated and adenoviral vectored vaccines in individuals with prior infection. They also shed light on the neutralization capacity against omicron variants and offer implications for the immunogenicity of the current vaccines deployed against the SARS-CoV-2 pandemic.

## 2. Materials and Methods

### 2.1. Study Design and Participants 

This prospective cohort study was conducted between 14 May and 9 December 2021 at the Center for Excellence in Clinical Virology, Chulalongkorn University, Bangkok. The participants were initially screened by physicians and nurse coordinators during the enrollment process. The inclusion criteria involved individuals previously infected with SARS-CoV-2 (identified through anti-nucleocapsid positivity (IgG) or a history of positive SARS-CoV-2 detection), those aged 18 years and older, and those without comorbidity or well-controlled comorbidities. The exclusion criteria encompassed participants who had received a different type of COVID-19 vaccine and those with severe medical conditions, such as compromised immune systems, malignancies, and autoimmune diseases.

**Figure 1:**
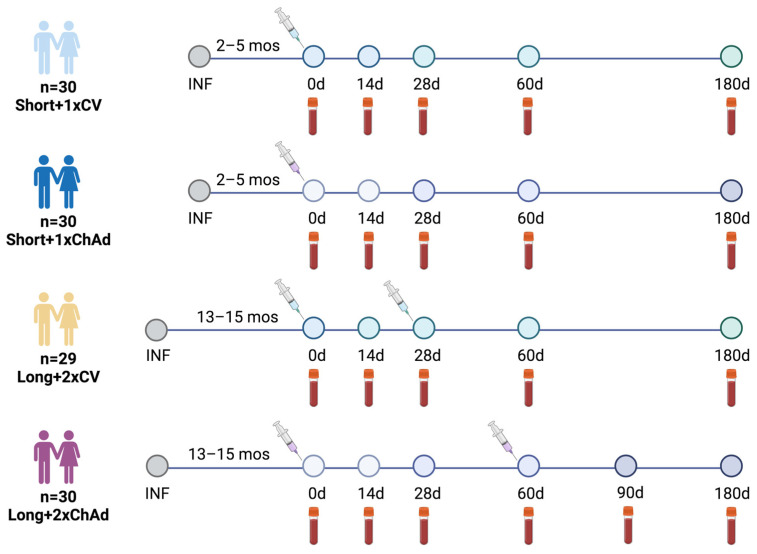
Schematic of vaccination timing and blood sampling. Previously infected individuals were categorized into four groups based on the time interval between the first positive SARS-CoV-2 detection and initial vaccination: short-interval (2–5 months) and long-interval (13–15 months), and type of vaccines: CoronaVac vaccine and ChAdOx1 vaccine. Participants in the short-interval group received a single dose of the vaccine, while those in the long-interval group were assigned to receive two doses of CoronaVac four weeks apart or two doses of ChAdOx1 eight weeks apart. Blood samples were collected at five time points (post-initial vaccination for all study groups): 0, 14, 28, 60, and 180 days. Additional blood samples were taken 90 days post-vaccination for the Long+2xChAd group. The figure was created with BioRender.com. CV = CoronaVac, ChAd = ChAdOx1 nCoV-19, INF = infection, and mos = months.

Participants were categorized based on the duration between their initial positive SARS-CoV-2 detection and the first vaccination, and they were divided into short- (2 to 5 months) and long-interval groups (13 to 15 months). The short-interval group comprised participants infected between 2 January and 13 April 2021 during the alpha predominant wave, and they were allocated to receive either a single dose of CoronaVac (referred to as Short+1xCV) or the ChAdOx1 nCoV-19 (referred to as Short+1xChAd) vaccine. Meanwhile, the long-interval group included individuals infected between 3 March and 4 April 2020 during the wild type predominant wave, and they were assigned to receive either two doses of CoronaVac (referred to as Long+2xCV) or the ChAdOx1 nCoV-19 (referred to as Long+2xChAd) vaccine. Within each group, participants were assigned to receive a vaccine based on convenience sampling and vaccine availability. Vaccination began on 14 May 2021, and the final blood sample was collected on 9 December 2021. The study design is illustrated in Figure 1.

Reactogenicity data were self-reported adverse events (AEs) collected seven days after the first vaccine doses. Blood samples were collected on days 0, 14, 28, 60, and 180 (6 months) after the first vaccination for the short-interval group. For the long-interval group, samples were collected on days 0, 14, and 28 after the first dose, and on days 28 and 120–150 (4 to 5 months) after the second dose of the vaccine. The study protocol adhered to the guidelines detailed in the Declaration of Helsinki and the Good Clinical Practice principles. Approvals were obtained from the Research Ethics Committee of the Faculty of Medicine, Chulalongkorn University (IRB numbers 192/64 and 281/64). This study has been registered with the Thai Clinical Trials Registry (TCTR20210319003 and TCTR20210520004). All participants provided written consent.

### 2.2. Study Vaccines

CoronaVac (Sinovac Life Sciences, Beijing, China) is an inactivated virus vaccine produced by cultivating the SARS-CoV-2 virus (CZ02 strain) in African green monkey kidney cells (Vero Cell). This is followed by inactivation using β-propiolactone and formaldehyde, as well as adsorption with aluminum hydroxide [2]. CoronaVac was administered within intervals of 21–28 days.

The ChAdOx1-vectored vaccine (ChAdOx1 nCoV-19) is a recombinant chimpanzee adenovirus-vectored vaccine that is replication-deficient and expresses the SARS-CoV-2 spike surface glycoprotein [3]. ChAdOx1 nCoV-19 was administered at 8 weeks apart.

### 2.3. Serological Testing

All serum samples underwent testing for anti-nucleocapsid (N) protein IgG and anti-receptor-binding domain (RBD) IgG antibodies against the ancestral strain using the commercially available automated ARCHITECT system (Abbott Diagnostics, Abbott Park, IL, USA) employing a chemiluminescent microparticle immunoassay (CMIA). The determination of anti-N IgG utilized the SARS-CoV-2 IgG assay (Abbott Diagnostics, Abbott Park, IL, USA) in accordance with the manufacturer’s instructions, with seropositivity defined as ≥1.4. The assessment of anti-RBD IgG employed the SARS-CoV-2 IgG II Quant assay (Abbott Diagnostics, Abbott Park, IL, USA) with a positive threshold set at equal to or greater than 50 AU/mL in accordance with the manufacturer’s guidelines. Conversion to binding antibody units per milliliter (BAU/mL) was conducted by multiplying the numerical AU/mL value by a factor of 0.142. An anti-RBD IgG result equal to or greater than 7.1 BAU/mL was considered positive.

### 2.4. Surrogate Virus Neutralization Tests (sVNT) for Pre- and Omicron Variants

Neutralizing activity against pre- and omicron variants induced by different vaccines was assessed using the cPass SAR-CoV-2 neutralizing antibody detection kit according to the manufacturer’s instructions (GenScript Biotech, Piscataway, NJ, USA). For this assay, serum samples obtained one month after the first and second vaccine doses were tested against recombinant SARS-CoV-2 RBD proteins of the wild type, B.1.1.7 (alpha), B.1.351 (beta), B.1.617.2 (delta), and B.1.1.529 (omicron BA.1) variants. Additionally, the sera collected six months after a single dose and 4–5 months after the second dose were tested against B.1.1.529 (omicron BA.1) using previously described methods [20]. Briefly, serum samples, along with positive and negative controls, were diluted at 1:10 and incubated with horseradish peroxidase-conjugated recombinant SARS-CoV-2 RBD proteins at 37 °C for 30 min. Subsequently, the reaction mixture was transferred to ELISA plates coated with human angiotensin-converting enzyme 2 proteins and incubated at 37 °C for 15 min. After washing, tetramethylbenzidine (TMB) solution was added, and the plate was incubated in the dark at room temperature for 15 min. After adding the stop solution, absorbance was promptly measured at 450 nm. Results were calculated as inhibition (%) = (1 − OD value of sample/average OD of negative control) × 100. Values above 30% indicated the presence of neutralizing antibodies.

### 2.5. Focus Reduction Neutralization Test (FRNT50)

Live SARS-CoV-2 neutralizing antibody titers against omicron BA.2 (EPI_ISL_11698090) subvariants were determined in a subset of serum samples collected one month and four to five months post-second dose using a 50% focus reduction neutralization test (FRNT50). The test involved assessing infected cell counts, as described previously [21]. Heat-inactivated serum samples underwent serial dilutions ranging from 1:10 to 1:7290. These diluted samples were then incubated with a live virus for 1 h at 37 °C. Afterward, the mixtures of the virus and sera were added to Vero cell monolayers in a 96-well plate and incubated for 2 h. The focus reduction percentage for each sample was calculated, and the half-maximal inhibitory concentration (IC50) was determined using PROBIT analysis from the SPSS package v23.0. In instances where no neutralization was observed, the FRNT50 was set at 10, which represents one dilution step below the lower limit of detection (dilution 1:20).

### 2.6. Quantification of Interferon-Gamma Response

In addition, the specific T-cell response to SARS-CoV-2 was assessed by measuring the total IFN-γ response in the whole blood following the manufacturer’s instructions (QuantiFERON, Qiagen, Hilden, Germany). Approximately 0.8–1.2 mL of heparinized whole blood was transferred to specialized blood collection tubes that contained two SARS-CoV-2 antigen tubes, a Mitogen tube (positive control), and a Nil tube (negative control). The blood collection tubes containing whole heparinized blood sample were then incubated for 24 h at 37 °C. The antigen tubes were coated with either S1 (RBD) peptides targeting CD4+ epitopes (Ag1) or S1+S2 peptides covering CD4+ and CD8+ epitopes (Ag2) from the ancestral strain. Following incubation, the tubes were centrifuged to collect the plasma. The plasma samples were then diluted at a 1:2 ratio with diluent and subjected to IFN-γ detection using an ELISA kit (Qiagen, cat. no. 626410) following the manufacturer’s guidelines. The concentration of IFN-γ was quantified based on an eight-point standard (ranging from 0.125 to 8 IU/mL) and calculated as IU/mL using QuantiFERON RD (v5.03) software. The final IFN-γ values were calculated by subtracting the value obtained from the Nil tube. A positive threshold was considered as IFN-γ (Ag1−Nil or Ag2−Nil) at ≥0.15 IU/mL and at ≥25% of Nil.

### 2.7. Statistical Analysis

The associations among the categorical variables were assessed using the chi-squared test or Fisher’s exact test, while the differences among the continuous variables were evaluated using one-way ANOVA with a Bonferroni adjustment. The geometric mean ratio (GMR) was calculated using a general linear model univariate analysis with log-transformed data, and it was adjusted for sex and age. A higher GMR indicated a slower decay rate. The fold decrease in neutralizing activity was calculated by comparing the results observed at one month with those at five to six months. Differences between matched paired samples were determined using paired sample *t*-tests or Wilcoxon matched-pairs signed-rank tests for nonparametric data. The comparisons of the differences between groups were conducted using analysis of covariance (ANCOVA) with a Bonferroni adjustment or Kruskal–Wallis tests with Dunn’s post hoc correction for nonparametric data. Furthermore, the data from vaccinated naïve individuals who received a two-dose regimen of CoronaVac or ChAdOx1, as has been previously reported [22,23,24], were obtained as comparators, and the baseline characteristics are provided in Tables S1 and S2. All statistical analyses were performed using IBM SPSS Statistics v23.0 (IBM Corp., Armonk, NY, USA) and GraphPad Prism v9.4.1 (GraphPad, San Diego, CA, USA). Statistical significance was considered at a *p*-value of <0.05.

## 3. Results

### 3.1. Study Participants

A total of 119 individuals previously infected with SARS-CoV-2 were recruited. The demographic characteristics of the study participants are presented in Table 1. The total participants included 56 females (47.1%) and 63 males (52.9%). The mean (SD) age of participants in the short-interval group immunized with one dose of CoronaVac (*n* = 30) was 37.6 (9.4) years, and those who received one dose of ChAdOx1 (*n* = 30) was 39.4 (8.9) years. The mean (SD) age of two-dose vaccinated CoronaVac (*n* = 29) and ChAdOx1 (*n* = 30) recipients in the long-interval group was 43.1 (8.3) and 44.5 (12.2) years, respectively. Significant differences were observed in age between the Short+1xCV and Long+2xChAd groups, as well as in the time interval at 60 and 180 days of follow-up, since the initial vaccination among these four groups. The reactogenicity data, which were collected as self-reported adverse events (AEs) following the first vaccine dose, are presented in Figure S1. Adverse events appeared to be more frequent after receiving ChAdOx1 compared to the CoronaVac vaccine in individuals with prior infection. These events included injection site pain, swelling, redness, fever, headache, myalgia, fatigue, and dizziness. However, no serious adverse events were observed.

At the 6-month visit, 18 participants from the Short+1xCV group, 13 from the Short+1xChAd group, 10 from the Long+2xCV group, and 1 from the Long+2xChAd group were lost to follow-up; most of them were either reinfected with SARS-CoV-2 or received other vaccines.

### 3.2. An Extended Interval between Infection and Vaccination Might Enhance the Antibody Response and Neutralizing Activities

**Figure 2:**
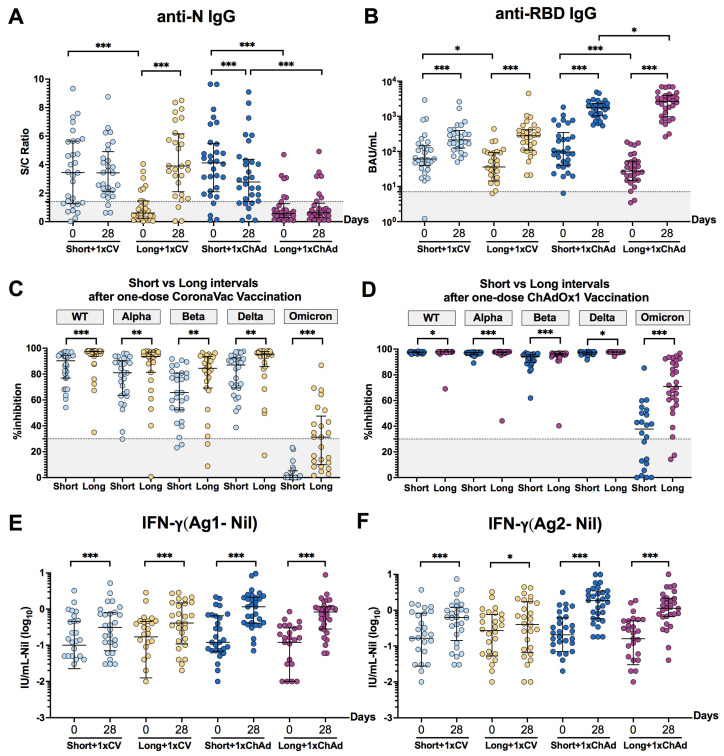
The impact of the time interval between natural infection and first vaccination on the immune response. The (**A**) anti-N IgG and (**B**) anti-RBD IgG, which involved neutralizing activities against pre-omicron variants and omicron (BA.1) after vaccination with (**C**) CoronaVac or (**D**) ChAdOx1 at 28 days post-vaccination, as well as the total IFN-γ responses to (**E**) Ag1 and (**F**) Ag2, were compared between the short- and long-interval groups. The median values with interquartile ranges are depicted as horizontal bars in panels (**A,C**–**F**). The geometric mean titers with 95% confidence intervals are shown as horizontal bars in panel (**B**). The dotted lines in panels (**A**–**D**) indicate cut-off values. Statistical differences between the sample collected at 0 and 28 days after a single dose vaccination were analyzed using Wilcoxon signed-rank tests in panels (**A**,**E**,**F**), while paired sample *t*-tests were conducted in panel (**B**). Differences between short- and long-interval groups were compared using Mann–Whitney U tests for panels (**A**,**C**–**F**), and independent sample *t*-tests were conducted in panel (**B**). Statistical significance was indicated as follows: * *p* < 0.05, ** *p* < 0.01, and *** *p* < 0.001.

To assess the impact of the time interval between infection and vaccination on the immune response, the results of antibody response, neutralization activities, and the level of total IFN-γ response on day 28 after a single dose vaccination were compared between the short- and long-interval groups. After a single dose of CoronaVac vaccination, participants in both short- and long-interval groups displayed a high titer of anti-N IgG and anti-RBD IgG on day 28, showing comparable levels despite their significantly different pre-existing antibody levels (Figure 2A,B). Conversely, after a single dose of ChAdOx1, the long-interval group exhibited significantly higher anti-RBD IgG levels compared to the short-interval group. Similarly, the neutralizing activities against pre-omicron variants—including wild type, alpha, beta, and delta, as well as the omicron variants—were significantly higher in the long-interval group than its counterpart, and this was observed in both vaccine types (Figure 2C,D). However, no significant difference was observed in the level of total IFN-γ responses after stimulation with SARS-CoV-2 antigens between the short- and long-interval groups following a single dose of CoronaVac vaccination or in the group vaccinated with the ChAdOx1 vaccine (Figure 2E,F). These results suggest that a longer interval between infection and vaccination could lead to a greater improvement in humoral immune response compared to a shorter interval. However, this effect was not observed in the T cell response.

### 3.3. Dynamic Changes and Waning of Anti-RBD IgG following CoronaVac or ChAdOx1 Vaccination in Individuals with Prior Infection

**Figure 3:**
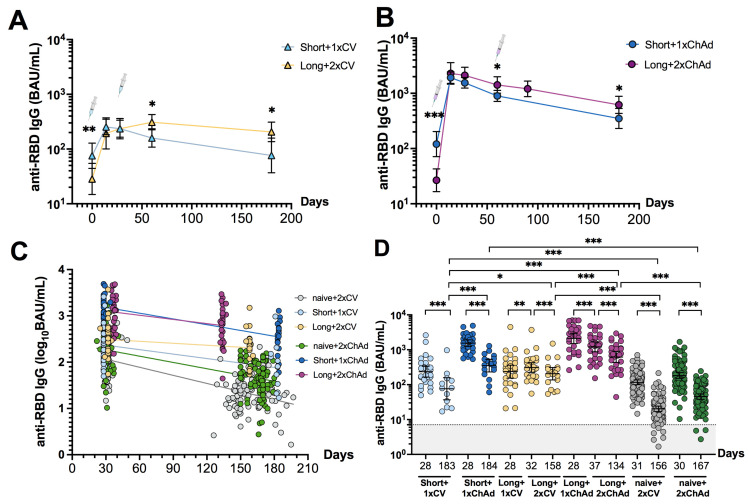
Dynamic changes in SARS-CoV-2 specific binding antibody response. Changes in anti-RBD IgG levels at different time points were compared (**A**) between the Short+1xCV and Long+2xCV groups and (**B**) between the Short+1xChAd and Long+2xChAd groups. (**C**) shows the decay of anti-RBD IgG levels in vaccinated individuals with and without previous SARS-CoV-2 infection. (**D**) shows the pairwise comparison in anti-RBD IgG levels between 1 month and 4–6 months following the first or two-dose vaccination. Geometric mean titers with 95% confidence intervals are shown as horizontal bars in panels (**A**,**B**,**D**). The dotted line indicates the cut-off value (panel (**D**)). The statistical differences in panels (**A**,**B**) were assessed using independent sample *t*-tests. The slopes of the fitted lines in panel (**C**) were estimated using linear regression analysis. The difference in panel (**D**) was assessed using paired sample *t*-tests for intra-group and one-way ANOVA with a Bonferroni adjustment for inter-group comparisons. The data on vaccinated naïve individuals are shown here for comparative reasons only. Statistical significance is indicated as follows: * *p* < 0.05, ** *p* < 0.01, and *** *p* < 0.001.

We examined the dynamic changes in anti-RBD IgG levels following one and two doses of different vaccines. After the second dose in the long-interval group, the antibody levels were significantly higher and more persistent compared to a single dose in the short-interval group (Figure 3A,B). The second dose of the CoronaVac vaccine led to a peak antibody response (GMT: 309.3 (224.6 to 425.9)), one that was higher than the peak after the first dose (GMT: 235.6 (153.4 to 361.9)); this differed from the ChAdOx1 vaccine, which induced a higher response after the first dose (GMT: 2114 (1510 to 2960)) and no significant increase after the second dose (GMT: 1205 (870.7 to 1668)). This suggests that either a single dose of a vector-based vaccine or two doses of an inactivated vaccine can elicit a robust immune response in recovered individuals.

To determine whether the decline rate of anti-RBD IgG differed between recovered individuals vaccinated with CoronaVac or ChAdOx1, we compared the slopes of fitted lines between 1 and 5–6 months after the first or second dose (Figure 3C). In the short-interval group, the estimated change in anti-RBD IgG levels (slope) was −0.0032 log10 BAU/mL per day for one dose of CoronaVac and −0.0041 log10 BAU/mL per day for one dose of ChAdOx1. In the long-interval group, the estimated decline rate was −0.0014 log10 BAU/mL per day for two doses of CoronaVac and −0.003 log10 BAU/mL per day for two doses of ChAdOx1. These results suggest a slower decline in anti-RBD IgG levels with CoronaVac compared to ChAdOx1 vaccination in recovered individuals.

We also compared the geometric mean ratio (GMR) of anti-RBD IgG kinetics between one month and five to six months after receiving the first or second dose. Overall, a significant decrease in anti-RBD IgG levels was observed in all groups at the later time point (Figure 3D). Moreover, ChAdOx1-vaccinated individuals maintained a higher titer of anti-RBD IgG levels compared to those vaccinated with CoronaVac. The GMR of the Short+1xCV group was 0.31 (0.23–0.42) and Short+1xChAd group was 0.2 (0.14–0.29). Meanwhile, the Long+2xCV group showed a GMR of 0.52 (0.46–0.59), and the Long+2xChAd group had a GMR of 0.51 (0.48–0.56) (Table S3). Recovered individuals who received at least one vaccine dose exhibited a slower decline in anti-RBD IgG levels compared to naïve individuals who received a two-dose regimen, except for the Short+1xChAd group. However, all of the individuals still had detectable anti-RBD IgG at six months post-vaccination.

### 3.4. Hybrid Immunity Induces Broader Neutralizing Activities and Persists for at Least 6 Months after Vaccination Compared to Solely Receiving Vaccination

**Figure 4:**
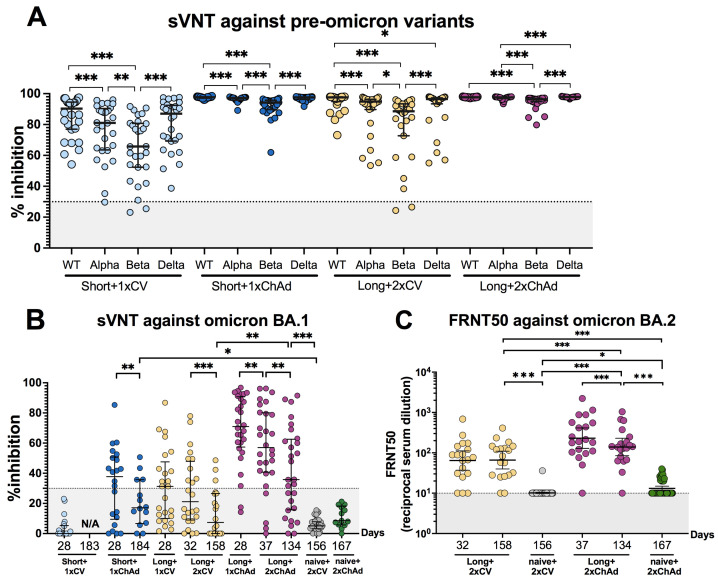
Neutralizing activity against SARS-CoV-2 pre-omicron and omicron variants. (**A**) Neutralizing activities against pre-omicron variants, including wild type, alpha (B.1.1.7), beta (B.1.352), and delta (B.1.617.2); these were assessed at 28 days after the first or two-dose vaccination using surrogate virus neutralization tests (sVNT). (**B**) Neutralizing activities against omicron BA.1 were compared between 1 and 5–6 months following the first or two-dose vaccination. (**C**) The live SARS-CoV-2 neutralizing antibody titers against omicron BA.2 assessed using a 50% focus reduction neutralization test (FRNT50) were compared between 1 and 4–5 months following the two-dose vaccination. Median values are depicted as horizontal bars with interquartile ranges in panels (**A**,**B**). Geometric mean titers with 95% confidence intervals are shown as horizontal bars in panel (**C**). The dashed line in panels (**A**–**C**) indicates the cut-off value. The statistical differences in panels (**A**,**B**) were assessed using the Friedman test with Dunn’s multiple comparisons test and Wilcoxon signed-rank tests, respectively. The differences in panel (**C**) were analyzed using the paired sample *t*-test for the intra-group and the independent sample *t*-test for inter-group comparisons. Statistical significance is indicated as follows: * *p* < 0.05, ** *p* < 0.01, and *** *p* < 0.001.

We conducted a surrogate virus neutralization test (sVNT) to determine neutralizing activity against pre-omicron variants (wild type, alpha, beta, and delta) at 28 days post-vaccination. After a single dose vaccination, neutralizing activities ranged from 65.8% to 90.3% in the Short+1xCV group and from 94.2% to 97.7% in the Short+1xChAd group (Table S4). At 28 days post-second dose, neutralizing activities ranged from 88.6% to 97.65% in the Long+2xCV group and from 96.4% to 97.7% in the Long+2xChAd group. The lowest neutralizing activity was observed against the beta variant, followed by alpha, delta, and wild type variants (Figure 4A).

To assess the neutralizing activity against the omicron BA.1 variant, serum samples were tested against the recombinant SARS-CoV-2 (omicron BA.1) RBD protein. At 28 days post-vaccination, 59.1% (13/22) of individuals in the Short+1xChAd group showed detectable neutralizing antibodies against the omicron variant. For the Long+2xCV group, 41.7% (10/24) showed seropositivity, while 85.7% (24/28) in the Long+2xChAd group demonstrated cross-reactive neutralization against omicron. Compared to infection-naïve vaccinated individuals, almost all study groups exhibited increased neutralizing activity against omicron, except for the Short+1xCV group.

To evaluate the decline in neutralizing activity against omicron, we calculated the fold decrease in neutralizing activity between one month and five to six months post-vaccination (Figure 4B). The Long+2xCV group exhibited the highest fold reduction in neutralizing activity (2.85-fold reduction), followed by a 2.21-fold drop in the Short+1xChAd group and a 1.59-fold decrease in the Long+2xChAd group. Moreover, the sera from individuals in the long-interval groups were selected to evaluate their ability to neutralize the live virus omicron BA.2 variant. The results between one month and four to five months after the second dose of vaccination were compared among recovered individuals who received vaccinations with either CoronaVac or ChAd (Figure 4C). No significant difference was observed between one month and five months after the second dose of CoronaVac, with geometric mean titers (GMTs) of 64.5 (37.1–111.9) and 66.1 (39.9–109.6), respectively. This result suggests the persistence of cross-reactive neutralizing antibodies against omicron. However, a significant drop in neutralizing antibody titers was found in the Long+2xChAd group, with GMTs decreasing from 230.8 after one month to 139.5 after four months following the second dose of the ChAd vaccination.

### 3.5. Decline of the Total T Cell IFN-γ Response after Vaccination in Individuals with Prior Infection

**Figure 5:**
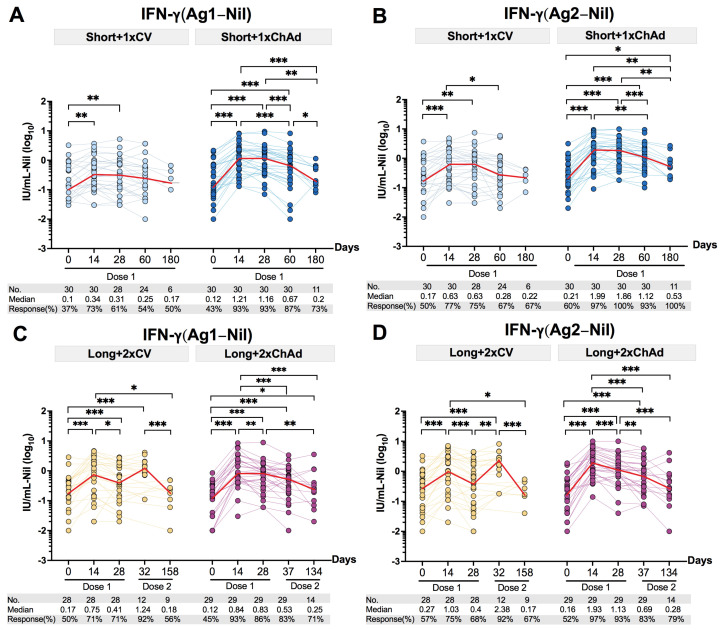
Dynamic changes in the total IFN-γ responses to SARS-CoV-2 antigens. Total IFN-γ responses to (**A**) Ag1 and (**B**) Ag2 were detected in those in the short-interval group vaccinated with a single dose of CoronaVac or ChAdOx1. Total IFN-γ responses to (**C**) Ag1 and (**D**) Ag2 were detected in those in the long-interval group with two-dose vaccination with CoronaVac or ChAdOx1. The median of the IFN-γ level is depicted by a red connecting line. The statistical differences in panels (**A**,**B**) were assessed using mixed-effects analysis with a Geisser–Greenhouse correction. Statistical significance is indicated as follows: * *p* < 0.05, ** *p* < 0.01, and *** *p* < 0.001.

To assess the dynamic changes in T cell responses, we measured the total IFN-γ response upon stimulation with CD4+ epitopes (Ag1), or with both CD4+ and CD8+ epitopes (Ag2), from the SARS-CoV-2 spike protein. Initially, nearly half of the participants in both the short- and long-interval groups displayed a reactive T cell response. Post-vaccination, the T cell responses peaked at 14 days but subsequently declined. Comparing the median IFN-γ response (Ag1) between one and six months post-vaccination revealed a 1.8-fold reduction in the Short+1xCV group and a 5.8-fold decrease in the Short+1xChAd (Figure 5A) group. Similarly, the median IFN-γ response reduction (Ag2) was lower in the Short+1xCV (2.9–fold decrease) group compared to the Short+1xChAd (3.5-fold reduction) group (Figure 5B). In the long-interval groups, a significant increase in T cell response was observed after the second dose of CoronaVac vaccine, while there was no notable elevation after the second dose of the ChAdOx1 vaccine.

Comparisons of the median IFN-γ response reduction (Ag1) between one and four to five months post-second dose vaccination revealed a 6.9-fold decrease in the Long+2xCV group and a 2.1-fold decrease in the Long+2xChAd group (Figure 5C). Similarly, assessing the median IFN-γ response reduction (Ag2) showed a higher reduction in the Long+2xCV group (14-fold) compared to the Long+2xChAd group (2.5-fold), indicating a faster reduction in the T cell response post-second dose of the CoronaVac vaccine (Figure 5D). Although the median IFN-γ response at 6 months post-vaccination reduced to levels similar to pre-vaccination, immunization in individuals with prior infection increased the number of individuals with a reactive T cell response in all groups. Therefore, vaccination with CoronaVac or ChAdOx1 vaccines provided a detectable benefit in the T cell response across all groups.

## 4. Discussion

This study examined the durability of hybrid immunity, and it explored both the humoral and T cell responses up to six months after CoronaVac or ChAdOx1 vaccination in previously infected participants with varying intervals between infection and first vaccination. We found both CoronaVac and ChAdOx1 vaccinations in previously infected individuals elicited higher antibody levels and enhanced neutralizing activities against pre-omicron and omicron variants when compared to infection-naïve individuals. This suggests that the enhanced response to inactivated and adenoviral vector-based vaccinations relies on pre-existing immunity, and this is consistent with earlier findings in mRNA vaccination [25,26]. Moreover, vaccinations after a longer interval (13 to 15 months) between infection and first vaccination induces higher neutralizing activity than a shorter interval (2 to 5 months). In addition, binding antibody levels and neutralizing activities against the omicron BA.1 and BA.2 variants persisted above pre-vaccination levels at six months post-vaccination, albeit they also showed a gradual waning. Although the total IFN-γ response declined to pre-vaccination levels by six months post-vaccination, a vaccination after infection benefits an increasing number of individuals with a reactive T cell response.

In the absence of immunization, we observed a sustained, albeit low-titer, presence of anti-RBD IgG for 13–15 months following SARS-CoV-2 infection, which aligns with a previous study [27]. The persistence of the antibody response was associated with a relatively stable number of RBD-specific memory B cells at least one year after infection [28]. Moreover, we found that nearly half of the individuals maintained reactive T cell responses for 13–15 months after infection. Consistent with a previous report, there was indications that a polyfunctionality and proliferation capacity of SARS-CoV-2-specific T cells persists at least 12 months post infection [29].

In line with our findings, several studies have indicated that hybrid immunity, which combines prior infection and mRNA vaccination, elicits robust antibody responses, often by displaying superior outcomes compared to vaccination in infection-naïve individuals [12,13,30,31]. Additionally, our study was extended to identify the effect of the hybrid antigen exposure interval on the magnitude of the immune response. Consistent with previous studies [25,32], a longer interval between infection and vaccination could enhance the level of antibody response and the strength of neutralization against SARS-CoV-2 variants. This finding could be partially explained by the increasing number of RBD-specific memory cells at 6 months compared to 1 month, which remain relatively stable at 12 months after infection [28]. Moreover, memory B cells continue to evolve even after 12 months post-infection, whereby they accumulate somatic mutations that increase neutralizing activity against SARS-CoV-2 mutants [28].

Consistent with a previous report [13], our results showed that a two-dose vaccination with inactivated vaccines or adenoviral vectors in recovered individuals provides cross-neutralization against both pre-omicron variants and the omicron variant, even though most of the participants had been infected with the wild type and alpha variant. This was supported by a gradual increase in memory B cell cross-binding to SARS-CoV-2 variants observed after vaccination in recovered individuals [33]. Furthermore, the inconsistent results regarding neutralization against omicron, which was observed through surrogate neutralizing assays and live virus neutralization following CoronaVac vaccination, might be explained by the distinct structural stabilization structure of spike proteins generated by inactivated (whole virus) and genetic vaccines (adenoviral-vectored vaccine) [34]. Moreover, the vaccine induces antibodies targeting other SARS-CoV-2 components, thereby potentially aiding in live virus neutralization, such as antibodies against the nucleoprotein [27].

The decline of the immune response over time and the ongoing evolution of virus variants raise concerns about the longevity of the protective immune response. Our results indicate that hybrid immunity provides a slower decline rate of antibody response and maintains broad neutralizing activities against omicron variants for up to six months. This result was supported by a real-world study [35], thus indicating that vaccination in previously infected individuals appears to enhance vaccine effectiveness, i.e., exceeding 90%, and extend immunity, and this is with no observed decline after more than 1 year post-infection or 6 months post-vaccination. Despite individuals who were previously infected and received two doses of CoronaVac showing a slower decline compared to ChAdOx1, their binding antibody levels and neutralizing activities remained at a low titer six months after vaccination [36]. Conversely, a single dose of ChAdOx1 exhibited robust antibody and neutralizing activity that was consistent with those reported in previous studies [14,37], while a second dose did not clearly enhance the immune response in individuals with prior infection. Similar results were found in mRNA vaccination, indicating that immunization with a single dose significantly boosted the expansion of pre-existing memory B cells in individuals with prior infection, while minimal changes in antibody response was observed after a second dose [11]. However, an alternative explanation could be the influence of anti-ChAd vector antibodies, which were reported to be well maintained for at least six months after vaccination [3]. Although a second dose of ChAd did not clearly increase antibody response, the advantage of a two-dose vaccination in terms of neutralization and slower decline was observed. This is consistent with reports that three antigen exposures, such as two vaccinations in convalescent individuals, could increase antibody avidity, thereby resulting in highly potent neutralization capacity against immune escape variants, including omicron, and maintenance for at least seven months [13].

The superiority of the T cell response in hybrid immunity was reported in prior infections following mRNA vaccination, and it was associated with an enhanced T follicular helper cell polarization of spike protein-specific CD4+ T cells, which likely enhanced IFN-γ production [30]. Our results have shown that vaccination with CoronaVac or ChAdOx1 could induce a high level of total T cell IFN-γ response in individuals with prior infection, indicating that spike-specific memory T cells are relatively sustained for more than six months after infection [38,39]. Although the increase in the median IFN-γ response was transient and returned to near pre-vaccination levels by 6 months post-vaccination, an increase in the number of individuals with a reactive T cell response was observed.

This study has several limitations. Initially, the number of participants at the six-month follow-up was relatively small, thus cautioning against broad generalizations of the results. Another limitation is the lack of data on hybrid immunity in vaccinated individuals who subsequently experience breakthrough infection. Our longitudinal cohort lacks infection-naïve individuals; however, we mitigated this limitation by obtaining data from our previous studies [22,23,24] for comparison, where we employed the same immunoassay to determine the humoral immune response. Additionally, the infecting sequence data were unavailable for participants with prior infection. Nonetheless, it was observed that the majority of recovered participants classified into the long-interval and short-interval groups were infected during the predominant periods of the wild type and alpha variants, respectively. Moreover, a previous study [40] found that the Abbott Diagnostics SARS-CoV-2 IgG assay was not very sensitive in detecting anti-N antibodies in individuals who had previously been infected with SARS-CoV-2 for an extended period. Therefore, caution is needed when interpreting the results. Furthermore, the results of neutralizing activity against pre-omicron variants, which were determined using the RBD-human ACE2 binding inhibition assay, reached the upper limit of detection. Additionally, our study only investigated spike-specific T cell responses, thus warranting further investigation to explore T cell responses against non-spike epitopes.

## 5. Conclusions

The findings suggest a priming role for infection-induced immunity and the boosting effect of CoronaVac or ChAdOx1 vaccination in previously infected individuals, thereby highlighting their durability in antibody and neutralization. Although the T cell response showed a transient increase, the persistent levels of total IFN-γ response at six months and an increased number of individuals with reactive T cell responses were observed. These findings provide valuable insights into the dynamic changes in the immune response triggered by inactivated and adenoviral vector vaccines among those with prior infection. Understanding the durability and broad effectiveness of immune responses induced by various vaccine platforms remains crucial as countries refine their vaccination strategies.

## Figures and Tables

**Table 1:** The demographics and characteristics of the participants previously infected with SARS-CoV-2 who were enrolled in this study. Participants were classified into four groups according to the duration between infection and first vaccination (short and long intervals) and type of vaccines (CoronaVac or ChAdOx1 nCoV-19). Participants with short and long intervals were assigned to receive a single dose and two doses of vaccines, respectively.

Characteristics	Total (*n* = 119)	Short ^a^+1xCV (*n* = 30)	Short ^a^+1xChAd (*n* = 30)	Long ^b^+2xCV (*n* = 29)	Long ^b^+2xChAd (*n* = 30)	*p*-Value
Age, years						0.027 ^c^
Mean (SD)	41.1 (10.1)	37.6 (9.4)	39.4 (8.9)	43.1 (8.3)	44.5 (12.2)	
Median (IQR)	39 (34–49)	35 (32–45)	38.5 (34–44)	43 (38–51)	39.5 (35–54)	
Sex						0.092 ^d^
Female, *n* (%)	56 (47.1%)	15 (50%)	13 (43.3%)	9 (31%)	19 (63.3%)	
Male, *n* (%)	63 (52.9%)	15 (50%)	17 (56.7%)	20 (69%)	11 (36.7%)	
BMI (kg m^−2^)						0.301 ^d^
BMI < 30	105 (88.2%)	26 (86.7%)	26 (86.7%)	24 (82.8%)	29 (96.7%)	
BMI ≥ 30	14 (11.8%)	4 (13.3%)	4 (13.3%)	5 (17.2%)	1 (3.3%)	
Comorbidities ^c^, *n* (%)	17 (14.3%)	2 (6.7%)	3 (10%)	6 (20.7%)	6 (20%)	0.282 ^d^
Symptomatic						0.075 ^d^
w pneumonia	36 (30.3%)	4 (13.3%)	11 (36.7%)	12 (41.4%)	9 (30%)	
w/o pneumonia	83 (69.7%)	26 (86.7%)	19 (63.3%)	17 (58.6%)	21 (70%)	
Interval between infection and first dose vaccination						
Median (IQR) days	N/A	74 (64–131)	63 (62–151)	425 (416–427)	426 (417–434)	<0.001 ^c^
Collection interval since first vaccination						
2nd visit (*n*) Median (IQR) days	(*n* = 119) 14	(*n* = 30) 14	(*n* = 30) 14	(*n* = 29) 14	(*n* = 30) 14	0.536 ^c^
3rd visit (*n*) Median (IQR) days	(*n* = 117) 28	(*n* = 28) 28	(*n* = 30) 28	(*n* = 29) 28	(*n* = 30) 28 (28–29)	0.075 ^c^
4th visit (*n*) Median (IQR) days	(*n* = 116) 62 (60–64)	(*n* = 28) 64 (63–65)	(*n* = 30) 64 (60–64)	(*n* = 28) 60	(*n* = 30) 62	<0.001 ^c^
5th visit (*n*) Median (IQR) days	N/A	N/A	N/A	N/A	(*n* = 30) 99 (99–100)	
Timing of 6-month visit Median (IQR) days	(*n* = 77) 186 (184–196)	(*n* = 12) 183 (181–183)	(*n* = 17) 184 (183–184)	(*n* = 19) 186	(*n* = 29) 196 (195–196)	<0.001 ^c^

^a^ Short interval refers to participants who had a period of 2–5 months between SARS-CoV-2 infection and their first vaccination. ^b^ Long interval refers to participants who had a period of 13–15 months between SARS-CoV-2 infection and their first vaccination. ^c^ The statistical analysis was assessed using one-way ANOVA with a Bonferroni adjustment. ^d^ The statistical analysis was evaluated using the chi-squared test or Fisher’s exact test. Abbreviation: CV, CoronaVac vaccine; ChAd, ChAdOx1 nCoV-19 vaccine; IQR, interquartile range; SD, standard deviation; BMI, body mass index; and N/A, not available.

## Data Availability

All data are available in this manuscript. Additional information can be obtained from the corresponding authors upon reasonable request.

## Supplementary Materials

The following supporting information can be downloaded at: https://www.mdpi.com/article/10.3390/vaccines12020180/s1, Figure S1: Forest plot of the solicited local and systemic adverse events within seven days after first dose vaccination with CoronaVac or ChAdOx1 in participants previously infected with SARS-CoV-2; Table S1: Baseline characteristics of naïve-infected individuals who received a two-dose of CoronaVac vaccine, as obtained from previous reports and used as the basis for comparison; Table S2: Baseline characteristics of naïve-infected individuals who received a two-dose of ChAdOx1 vaccine, as obtained from previous reports and used as a basis for comparison; Table S3. The geometric mean titers (GMTs) and geometric mean ratios (GMRs) of anti-RBD IgG (BAU/mL) compared between day 28 and day 130 to 180 after first or second dose vaccination: intra-group and inter-group comparisons; Table S4: Neutralizing activity against pre-omicron and omicron variants measured using a surrogate virus neutralization test (sVNT).
