# Supplementary material for: Long-Term Dynamic Changes in Hybrid Immunity over Six Months after Inactivated and Adenoviral Vector Vaccination in Individuals with Previous SARS-CoV-2 Infection
Source: Vaccines (Basel). 2024 Feb 10;12(2):180. doi: 10.3390/vaccines12020180 (PMC10891631; doi:10.3390/vaccines12020180)
Supplement: Supplementary file 1 [file vaccines-12-00180-s001.zip › vaccines-2837969-supplementary.pdf]

Supplementary Information

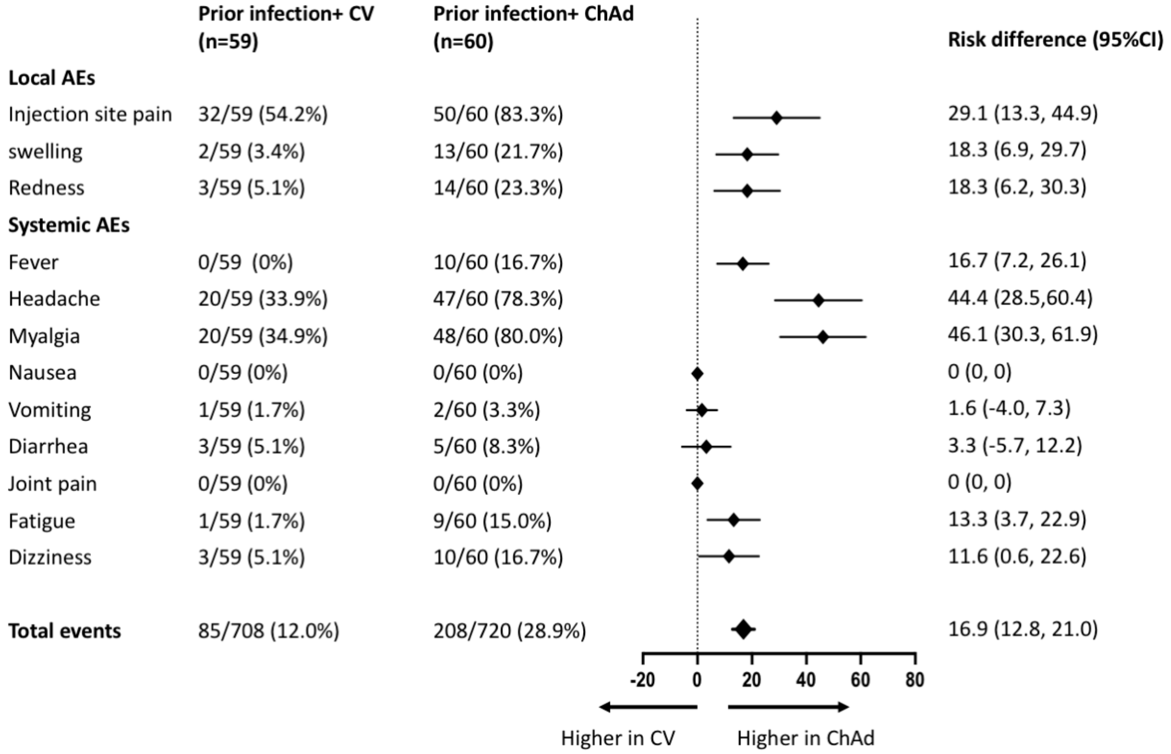

Supplementary Figure S1. Forest Plot displayed solicited local and systemic adverse events within seven days after first dose vaccination with CoronaVac or ChAdOx1 in participants previously infected with SARS-CoV-2. Prior infection was defined as individuals with short and long intervals between infection and vaccination. AEs, adverse events; CV, CoronaVac; ChAd, ChAdOx1 nCoV-19; CI, confidence interval**.**

Supplementary Table S1. Baseline characteristics of naïve-infected individuals who received a two-dose of CoronaVac vaccine were obtained from previous reports and used as comparators. They were then compared to those of individuals with prior infection who received the CoronaVac vaccine.

Characteristics	Short[a]+1xCV post 1st dose (n=30)	Long[b]+ 2xCV post 2nd dose (n=29)	Naïve+2xCV 1 month post 2nd dose (n=89)	Naïve +2xCV 6 months post 2nd dose (n=88)	*p*-value
Age, years<br>Mean (SD)<br>Median (IQR)	37.6 (9.4)<br>35 (32–45)	43.1 (8.3)<br>43 (38–51)	42.6 (9.6)<br>42 (36–49)	40 (9.1)<br>40 (34–47)	0.027[c]
Sex<br>Female, n (%)<br>Male, n (%)	15 (50%)<br>15 (50%)	9 (31%)<br>20 (69%)	40 (44.9%)<br>49 (55.1%)	49 (55.7%)<br>39 (44.3%)	0.121[d]
Timing of 1 month visit<br>Median (IQR) days	28	32	31 (30–32)	N/A	<0.001[d]

Timing of 6 months visit<br>Median (IQR) days	183 (181–183)	158	N/A	156 (147–168)	<0.001[d]

[a] Short-interval refers to participants who had a period of 2–5 months between SARS-CoV-2 infection and their first vaccination. [b] Long-interval refers to participants who had a period of 13–15 months between SARS-CoV-2 infection and their first vaccination. [c] Comparison between the groups was assessed using One-way ANOVA with Bonferroni adjustment. [d] Comparison between the groups was evaluated using the Chi-squared test or Fisher's exact test. Abbreviations: CV, CoronaVac; IQR, interquartile range; SD, standard deviation; N/A, no data available.

Supplementary Table S2. Baseline characteristics of naïve-infected individuals who received a two-dose of ChAdOx1 vaccine were obtained from previous reports and used as comparators. They were then compared to those of individuals with prior infection who received the ChAdOx1 vaccine.

Characteristics	Short[a]+1xChAd post 1st dose (n=30)	Long[b]+ 2xChAd post 2nd dose (n=29)	Naïve+2xChAd 1 month post 2nd dose (n=88)	Naïve +2xChAd 6 months post 2nd dose (n=87)	*p*-value
Age, years<br>Mean (SD)<br>Median (IQR)	 39.4 (8.9)<br>38.5 (34–44)	 44.5 (12.2)<br>39.5 (35–54)	 48 (13)<br>47 (39–58)	 53.9 (12.5)<br>56 (45–64)	<0.001[c]
Sex<br>Female, n (%)<br>Male, n (%)	 13 (43.3%)<br>17 (56.7%)	 19 (63.3%)<br>11 (36.7%)	 49 (55.7%)<br>39 (44.3%)	 48 (55.2%)<br>39 (44.8%)	0.474[d]
Timing of 1 month visit<br>Median (IQR) days	28	37(37–38)	30 (30–31)	N/A	<0.001[c]
Timing of 6 months visit<br>Median (IQR) days	184 (183–184)	134 (133–134)	N/A	167 (161–170)	<0.001[c]

[a] Short-interval refers to participants who had a period of 2–5 months between SARS-CoV-2 infection and their first vaccination.
[b] Long-interval refers to participants who had a period of 13–15 months between SARS-CoV-2 infection and their first vaccination.
[c] Comparison between the groups was assessed using One-way ANOVA with Bonferroni adjustment. [d] Comparison between the groups was evaluated using the Chi-squared test or Fisher's exact test. Abbreviations: CV, CoronaVac; IQR, interquartile range; SD, standard deviation; N/A, no data available.

Supplementary Table S3. Geometric Mean Titers (GMTs) and Geometric Mean Ratios (GMRs) of anti–RBD IgG (BAU/mL) compared between Day 28 and Day 130 to 180 after first or second dose vaccination: Intra–Group and Inter–Group Comparisons.

CoronaVac vaccine	Naïve +2xCV	Short+1xCV	Long+2xCV
Day 28<br>GMT (95%CI)<br>GMR<br>(95%CI, *p*-value)	 116.5 (99.6–136.3)<br>ref	 235.4 (164.8–336.2)<br>2.02<br>(1.33–3.07, *p* <0.001) [a]	 309.3(224.6–425.9)<br>2.65<br>(1.75–4.04, *p* <0.001) [a]
Day 130–180<br>GMT (95%CI)<br>GMR<br>(95%CI, *p*-value)	 20.3 (17.0–24.3)<br>ref	 76.4 (36.9–158.5)<br>3.77<br>(1.95–7.28, *p* <0.001) [a]	 205.6 (136.4–310.0)<br>10.14<br>(5.89–17.42, *p* <0.001) [a]

Day 130–180/Day 28<br>GMR<br>(95%CI, *p*-value)	0.17<br>(0.14–0.22, *p*<0.001) [c]	0.31<br>(0.23–0.42, *p* <0.001) [b]	0.52<br>(0.46–0.59, *p* <0.001) [b]
**ChAdOx1 vaccine**	**Naïve +2xChAd**	**Short+1xChAd**	**Long+2xChAd**
Day 28<br>GMT (95%CI)<br>GMR<br>(95%CI, *p*-value)	182.4 (150.9–220.4)<br>ref	1549 (1245–1926)<br>8.49<br>(5.53–13.03, *p* <0.001) [a]	1205 (870.7–1668)<br>6.61<br>(4.31–10.14, *p* <0.001) [a]
Day 130– 180,<br>GMT (95%CI)<br>GMR<br>(95%CI, *p*-value)	45.4 (37.8–54.4)<br>ref	350.5 (231–531.8)<br>7.73<br>(4.43–13.49, *p* <0.001) [a]	618.8 (432.8–884.6)<br>13.65<br>(8.69–21.43, *p* <0.001) [a]
Day 130–180/Day 28<br>GMR<br>(95%CI, *p*-value)	0.25<br>(0.19–0.32, *p*<0.001) [c]	0.20<br>(0.14–0.29, *p* <0.001) [b]	0.51<br>(0.48–0.56, *p* <0.001) [b]

[a] The statistical analysis was performed using general linear model (GLM) univariate analysis with the naïve infection group as reference. [b] The statistical analysis was evaluated using paired sample t–tests.
[c] The statistical analysis was evaluated using independent sample *t*-tests. Abbreviation: CV, CoronaVac vaccine; ChAd, ChAdOx1 nCoV-19; 95%CI, 95% Confidence Interval.

Supplementary Table S4. Neutralizing activity against pre–omicron and omicron variants was measured using a surrogate virus neutralization test (sVNT).

**Groups**	**Short+1xCV (n=30)**	**Short+1xChAd (n=30)**	**Long+2xCV (n=29)**	**Long+2xChAd (n=30)**
**sVNT-wild type**				
Day 28 (1st dose)<br>Median (IQR)	90.3 (77–94.5)	97.7 (97.1–97.9)	97.2 (89.5–97.8)	97.9 (97.8–98)
n	28	30	28	30
Day 28 (2nd dose)<br>Median (IQR)	N/A	N/A	97.6 (94.9–97.9)	97.7 (97.6–97.8)
n	N/A	N/A	27	29
**sVNT-alpha**				
Day 28 (1st dose)<br>Median (IQR)	81.1 (63.6–90.4)	97.2 (95.8–97.5)	93.5 (76.8–96.9)	97.8 (97.3–97.9)
n	28	30	28	30
Day 28 (2nd dose)<br>Median (IQR)	N/A	N/A	94.9 (89.7–96.4)	97.6 (96.9–97.8)

n	N/A	N/A	27	29
**sVNT-beta**				
Day 28 (1st dose) Median (IQR)	65.8 (52.4–80.8)	94.2 (89.7–95.7)	83.9 (67.8–93.6)	96.2 (95.4–96.6)
n	28	30	28	30
Day 28 (2nd dose) Median (IQR)	N/A	N/A	88.6 (72.7–93.4)	96.4 (94.9–97.1)
n	N/A	N/A	27	29
**sVNT-delta**				
Day 28 (1st dose) Median (IQR)	87.1(69.3–92.4)	97.6 (96.3–97.8)	95.1 (84.5–97.4)	97.8 (97.6–97.9)
n	28	30	28	30
Day 28 (2nd dose) Median (IQR)	N/A	N/A	96.5 (93.4–97.3)	97.8 (97.7–98)
n	N/A	N/A	27	29
**sVNT-omicron BA.1**				
Day 28 (1st dose) Median (IQR)	0 (0–5.25)	37.8 (9.5–50.8)	N/A	N/A
n	22	22	N/A	N/A
Day 28 (2nd dose) Median (IQR)	N/A	N/A	21.1 (9.0–47.9)	57 (40.7–80.1)
n	N/A	N/A	24	28

Abbreviations: ChAd, ChAdOx1 nCoV-19; CV, CoronaVac; IQR, interquartile range; SD, standard deviation; N/A, no data available.
